# Gas Outburst Warning Method in Driving Faces: Enhanced Methodology through Optuna Optimization, Adaptive Normalization, and Transformer Framework

**DOI:** 10.3390/s24103150

**Published:** 2024-05-15

**Authors:** Zhenguo Yan, Zhixin Qin, Jingdao Fan, Yuxin Huang, Yanping Wang, Jinglong Zhang, Longcheng Zhang, Yuqi Cao

**Affiliations:** College of Safety Science and Engineering, Xi’an University of Science and Technology, Xi’an 710054, China; 23220089060@stu.xust.edu.cn (Z.Q.); fanjd@126.com (J.F.); 20120089017@stu.xust.edu.cn (Y.H.); wang_yanping@xust.cn (Y.W.); 22220226186@stu.xust.edu.cn (J.Z.); 15591557109@163.com (L.Z.); 19403070424@stu.xust.edu.cn (Y.C.)

**Keywords:** transformer, PersistAD, adaptive normalization, VMD, dimensional analysis

## Abstract

Addressing common challenges such as limited indicators, poor adaptability, and imprecise modeling in gas pre-warning systems for driving faces, this study proposes a hybrid predictive and pre-warning model grounded in time-series analysis. The aim is to tackle the effects of broad application across diverse mines and insufficient data on warning accuracy. Firstly, we introduce an adaptive normalization (AN) model for standardizing gas sequence data, prioritizing recent information to better capture the time-series characteristics of gas readings. Coupled with the Gated Recurrent Unit (GRU) model, AN demonstrates superior forecasting performance compared to other standardization techniques. Next, Ensemble Empirical Mode Decomposition (EEMD) is used for feature extraction, guiding the selection of the Variational Mode Decomposition (VMD) order. Minimal decomposition errors validate the efficacy of this approach. Furthermore, enhancements to the transformer framework are made to manage non-linearities, overcome gradient vanishing, and effectively analyze long time-series sequences. To boost versatility across different mining scenarios, the Optuna framework facilitates multiparameter optimization, with xgbRegressor employed for accurate error assessment. Predictive outputs are benchmarked against Recurrent Neural Networks (RNN), GRU, Long Short-Term Memory (LSTM), and Bidirectional LSTM (BiLSTM), where the hybrid model achieves an R-squared value of 0.980975 and a Mean Absolute Error (MAE) of 0.000149, highlighting its top performance. To cope with data scarcity, bootstrapping is applied to estimate the confidence intervals of the hybrid model. Dimensional analysis aids in creating real-time, relative gas emission metrics, while persistent anomaly detection monitors sudden time-series spikes, enabling unsupervised early alerts for gas bursts. This model demonstrates strong predictive prowess and effective pre-warning capabilities, offering technological reinforcement for advancing intelligent coal mine operations.

## 1. Introduction

Preventing outburst disasters during coal mine driving is paramount to ensuring safe production. As mining depths increase, monitoring methane emissions and preventing coal and gas outbursts have become integral to mine design and operation. Countries such as Australia, China, Germany, and Russia possess extensive expertise in these areas. However, incidents of gas outbursts and subsequent chain reactions leading to dust explosions and disruptions in airflow continue to raise significant concerns. For example, tragic gas explosions occurred in Chinese coal mines in 2022, resulting in the loss of 21 lives across six fatal incidents, along with substantial property damage and casualties. Historical records show that between 1895 and 2017, Australia experienced 878 coal and gas outburst events [1,2]. In response to these challenges, advancements in artificial intelligence, including the utilization of large-scale models like ChatGPT, have been integrated into coal and gas outburst prediction efforts. However, key issues persist, notably the scarcity of sophisticated data modeling approaches, the complexity of representing interdependencies within datasets, and the limited interpretability of employed algorithms. Given the variability in gas emission characteristics across different mining regions, there is a pressing need to establish time-series models with enhanced predictive capabilities. This requires the formulation of rational indicators tailored to these unique regional variations, which can then be effectively implemented within gas outburst warning systems at driving faces of diverse mines. Such endeavors aim to elevate overall safety standards and mitigate the hazards associated with coal and gas outbursts.

Traditional forecasting approaches for coal and gas outbursts at mine workings pivot on three key elements: geological stresses, gas characteristics, and coal properties. Predictive methods are classified into single indicators, such as gas expansion energy, and composite indicators like the R, D, and K indices. Time-series gas concentration monitoring and machine learning algorithm implementations are also prevalent, along with surveillance methods integrating electromagnetic radiation, acoustic emissions, and microseismic monitoring [3].

Given the cyclical, stochastic, trending, and seasonal nature of gas emission datasets from mine workings, researchers have extensively explored coal mine gas emission data for anticipating outbursts, conducting time-series volume forecasts. Scholars worldwide have significantly advanced gas pre-warning research through exhaustive studies. Works by Anani and O, among others [4], emphasize the importance of input parameters in forecasting. The Outburst Danger Index (Ww), introduced by Dreger and Celary [5], provides a potent new tool for hazard assessment. Noferesti and Khakshour’s Phase2 simulations underscored the influence of parameters like cohesion and internal friction angle on coal strength [6].

Studies such as those by Fan, Li, and colleagues [7] analyzed interrelations among key elements in the outburst dynamic system, crucial for prevention measures. Wold, Connell, Connell, L.D., and others [8] investigated the relationship between outbursts and geological structures in Australian mines using Monte Carlo simulations for risk analysis. Zhang, Song, and team [9] proposed a multidimensional LSTM model for gas prediction, enhancing turning point accuracy. Chen, Wang, and others [10] developed a dynamic gas emission prediction model with enhanced precision through consideration of multiple influencers. Yu, Yang, and associates [11] applied rock engineering system theory and devised a cloud chart-based prediction scheme. Shi, Zhang, and co-authors [12] proposed a hybrid deep learning framework for real-time prediction of explosion overpressure using sparse data. Nie, Wang, and others [13] achieved a 96% accuracy in predicting gas explosion sites via confidence criteria with the F-SPA model. Shao, Chen, and collaborators [14] addressed data loss in gas prediction through optimized Extreme Learning Machines. Ji, Shi, and their team [15,16,17] used HDAIA and HPO algorithms for indicator correlation and BiLSTM optimization, respectively. Zhu, Zheng, and others [18] employed a systematic screening and appraisal method for outburst evaluation via a GA-BP neural network model. Hu, Shi, and others [19] found moisture content reduction to lower outburst intensity. Lastly, Agrawal, Durucan, and colleagues [20] adopted a probabilistic risk assessment framework, simulating 10,000 scenarios via Monte Carlo for longwall mining outburst forecasts. These researchers have collectively amassed a wealth of knowledge through theoretical calculations, numerical simulations, and computational experiments in the realm of gas outbursts and predictive strategies.

Nonetheless, practical engineering encounters constraints, namely a scarcity of data pertinent to driving faces, rendering machine learning methodologies less adaptable for forecasting gas emission volumes and hindering their universal application across disparate mining sites. Furthermore, these methodologies often overlook the variation in gas emission traits under differing operational conditions. In light of these considerations, this study undertakes an exhaustive examination of the intricate interrelationships within gas emission volume datasets. It adopts adaptive normalization techniques and refines the transformer architecture, harnessing Optuna for dynamic parameter tuning, and employs dimensional analysis in the creation of informative indicators. This comprehensive strategy is geared towards accurately forecasting gas emissions in driving scenarios and meticulously dissecting coal and gas outburst warning signals, with the overarching objective of devising a gas warning model distinguished by heightened adaptability and substantial automation. The ultimate intention is to furnish a tool that navigates the complexities of diverse mining environments with precision and efficiency, thereby augmenting safety protocols in coal extraction processes.

## 2. Materials and Methods

Central to this paper is the proposition of a time-series hybrid prediction model and an accompanying warning methodology. This time-series model integrates historical gas emission data, leveraging adaptive normalization and an enhanced transformer structure to proficiently extract temporal features from gas emission sequences. The core of the time-series hybrid prediction model rests upon the adaptive normalization (AN) technique for data normalization. Compared to conventional normalization approaches, AN strategically assigns weights to data points at varied timestamps, facilitating superior standardization of time-series data [21]. Following this, the Ensemble Empirical Mode Decomposition (EEMD) coupled with Variational Mode Decomposition (VMD) [22] is employed for data dissection, complemented by advancements to the transformer architecture. These enhancements couple the transformer’s potent feature extraction capabilities in extended time series with the integration of multilayer perceptron (MLP) for mapping prediction outputs, amplifying the model’s efficacy. Optimization of model parameters is facilitated through the Optuna framework, a tool adept at refining multiparameter models [23,24,25]. Subsequently, the xgbRegressor (Extreme Gradient Boosting Regressor) is deployed to gauge discrepancies between the original gas concentration sequences and predicted outcomes, with the specific version being XGBoost 2.0.3, thereby achieving robust time-series predictions. Building upon these foundations, Bootstrap sampling is utilized to ascertain confidence intervals for the hybrid model’s predictive outcomes. Moreover, an innovative unsupervised learning strategy for pinpointing anomalous segments in gas concentration data is put forth, utilizing dimensional analysis in the construction of indicative metrics. The Persist Anomaly Detection (PersistAD) methodology is then implemented to surveil these anomalies in gas concentration trends, furnishing a vital safety net for mining operations.

**Figure 1:**
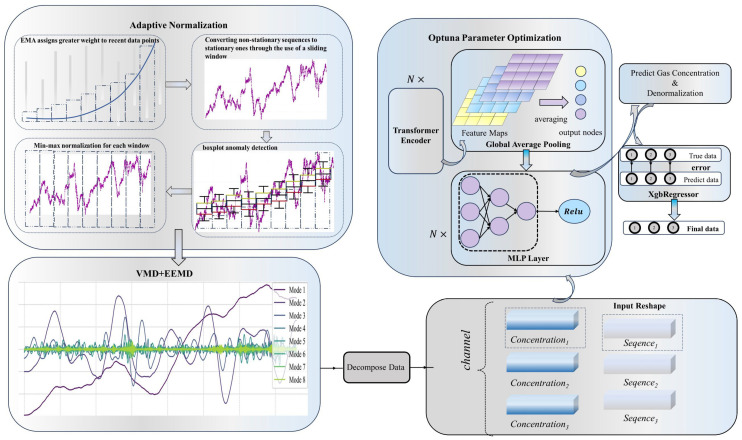
The overall framework for gas concentration prediction.

Figure 1 illustrates the comprehensive framework for predicting gas concentration levels. The purple line in the figure represents the original data. Initially, data undergo preprocessing via adaptive normalization. This process involves sequentially assigning greater weight to proximate points using EMA, transforming non-stationary sequences into stationary ones, identifying and excluding outliers in the data through boxplot analysis, and finally normalizing the dataset. Normalized data are subsequently subjected to an automated decomposition procedure employing the combined approach of Variational Mode Decomposition (VMD) and Ensemble Empirical Mode Decomposition (EEMD). The decomposed data channels are then channeled into an existing machine learning architecture, where meticulous parameter tuning is conducted using Optuna. This portion of the framework incorporates a transformer encoder, followed by global average pooling and interfaced with a multilayer perceptron (MLP) layer. The processed outputs are subjected to concentration-specific training, with a denormalization step incorporated to revert predictions to their original scale. The outcomes of this training phase necessitate comparison against actual data using the xgboost regressor (xgbRegressor), culminating in the derivation of final, refined predictions. In the xgbRegressor process, the numbers 1, 2, 3 in the gray circles represent consecutive moments.

### 2.1. Adaptive Normalization Data Standardization

Adaptive normalization (AN) [21], tailored for non-stationary heteroscedastic time-series data, constitutes a methodology surpassing conventional standardization techniques in accurately unraveling intrinsic time-series correlations. Its implementation unfolds through three successive phases: First, a transformation is enacted on the non-stationary time series, rendering it stationary by computing moving averages across non-overlapping sliding windows. This adjustment fosters a more stationary profile within the sequence. Secondly, outliers are identified and excised from the dataset, leveraging the boxplot methodology as the diagnostic tool to ensure data integrity. Lastly, leveraging comprehensive dataset metrics—the global extremities, namely the maximum and minimum values—a min–max normalization is universally applied across all delineated sliding windows. This maneuver harmonizes the scales, priming the data for analysis. Upon conclusion of the predictive phase, a restorative inverse normalization process is enacted, reinstating the original magnitude context to the forecasted data points, thereby preserving interpretative coherence with the initial dataset.

In the realm of gas emission data collected at driving faces, the data exhibit a notable bias towards recent times, with its influence waning as the timeframe extends. Implementing the Exponential Moving Average (EMA) approach for calculating the moving average effectively captures these temporal features by assigning greater weight to more recent data points, thereby encapsulating the dynamics of the near-term environment at these driving faces.

(1) $${EMA}_{1}=X_{1}$$

(2) $${EMA}_{t}=\alpha \times X_{t}+(1-\alpha )\times {EMA}_{t-1}$$

(3) α=2/(k+1)

In Equation (1), the initial EMA (${EMA}_{1}$) is calculated by taking the first moment’s actual data ($X_{1}$). In Equation (2), ${EMA}_{t}$ represents the EMA value at time t. $X_{t}$ is the original data value at time t. α is the smoothing factor, indicating the weight of historical data, typically ranging between 0 and 1. A higher α value indicates a greater influence of the current value. ${EMA}_{t-1}$ is the EMA value at time t−1. In Equation (3), k denotes the order of the EMA method, controlling the degree of responsiveness of EMA to new data. A smaller k value indicates a faster response of EMA to new data.

In Equation (1), initially, $X_{1}$, representing the data at the first time point, is taken as ${EMA}_{1}$. In Equation (2), ${EMA}_{t}$ represents the EMA value at time t, where $X_{t}$ is the original data value at time t. α is the smoothing factor, denoting the weight of historical data, typically ranging between 0 and 1. A higher α value indicates a greater influence of the current value. ${EMA}_{t-1}$ represents the EMA value at time (t−1). In Equation (3), k denotes the order of the EMA method, controlling the degree of EMA’s responsiveness to new data. A smaller k value implies faster responsiveness to new data. Equation (1) effectively transforms non-stationary time series into stationary ones.

Upon completion of the Exponential Moving Average (EMA) computation, the derived EMA sequence serves as the foundation for generating a stationary series. This process entails employing successive, non-overlapping sliding windows that partition the EMA sequence, ensuring that each window provides an independent glimpse into the statistical stability of the driving faces’ conditions over discrete periods.

(4) $$R=[r_{1},r_{2},\ldots ,r_{n-\omega +1}]$$

(5) $${r(i)}_{j}={X(k)}_{i+j-1}$$

In Equation (4), R represents the sliding window sequence, and ω represents the length of the sliding window. In Equation (5), r(i) represents a sliding window of length ω, where i is the starting position of the sliding window. X(k) represents the moving average sequence with a length of n−k+1.

In the AN data standardization process, following the aforementioned calculations, outlier values within each sliding window are identified using the boxplot method and subsequently removed. The optimal value for the moving average line is evaluated by assessing its fit from 1 to the size of the disjoint sliding windows. Once these steps are completed, the global minimum and maximum values are computed, and normalization is performed on the values within each sliding window, completing the adaptive normalization process.

### 2.2. Variational Mode Decomposition

The Variational Mode Decomposition (VMD) model represents a non-iterative signal processing technique that iteratively seeks the optimal representation through variational modes. It parses time-series information into a sequence of Intrinsic Mode Functions (IMFs), each characterized by a finite spectral spread, with the unique capability to iteratively refine the central frequencies and bandwidths tailored to each IMF. Conversely, Ensemble Empirical Mode Decomposition (EEMD) introduces an adaptive means to establish the hierarchical arrangement of these modes, albeit encountering the challenge of mode mixing, where signal components intertwine. To reconcile these strengths and limitations, a synergistic approach combines the EEMD’s mode ordering prowess with VMD’s precision. By first applying EEMD to decompose the signal and subsequently guiding VMD with the inferred mode sequence, the challenge of mode ordering typically encountered in VMD is mitigated effectively. This integrated methodology capitalizes on the complementary abilities of both algorithms, optimizing the separation and analysis of complex time-series data.

(6) $$u_{k}\left(t\right)=A_{k}\left(t\right)cos[\varphi_{k}(t)]$$

(7) $$\left\{\begin{array}{c} \underset{\left\{u_{k}\right\}\{\omega_{k}\}}{\min}\left\{\sum_{k=1}^{K}{\left\|\partial_{t}\left[\left(\delta (t)+\frac{j}{\pi t}\right)u_{k}(t)\right]e^{-j\omega_{k}(t)}\right\|}_{2}^{2}\right\}\\ s.t.\;\sum_{k=1}^{K}u_{k}\left(t\right)=f(t) \end{array}\right.$$

In Equation (6), $A_{k}\left(t\right)$ represents the amplitude of $u_{k}\left(t\right)$, and $\varphi_{k}\left(t\right)$ represents the phase angle of $u_{k}\left(t\right)$ in radians. In Equation (7), $\omega_{k}\left(t\right)$ represents the angular frequency of $u_{k}\left(t\right)$, rad/s, where k varies from 1 to K. δ(t) denotes the Dirac delta function. K represents the number of IMFs, and k ranges from 1 to K.

Introduce the penalty factor α and Lagrange multiplier λ(t) to construct the Lagrangian function as Equation (8).

(8) $$L\left\{u_{k}\right\},\left\{\omega_{k}\right\},\lambda \left(t\right)=\;\alpha \sum_{k=1}^{K}{\left\|\partial_{t}\left[\left(\delta (t)+\frac{j}{\pi t}\right)u_{k}(t)\right]e^{-j\omega_{k}(t)}\right\|}^{2}+\;{\left\|f\left(t\right)-\sum_{k=1}^{K}u_{k}(t)\right\|}_{2}^{2}+\langle \lambda \left(t\right),f\left(t\right)-\sum_{k=1}^{K}u_{k}(t)\rangle$$

Finally, the saddle point of Equation (8) is solved using alternating directions, and the results are transformed from the time domain to the frequency domain, obtaining the frequency domain formulas for each modal component as Equations (9) and (10).

(9) $$U_{k}^{n+1}\left(\omega \right)=\frac{F\left(\omega \right)-\sum_{k\neq i}U_{i}\left(\omega \right)+\frac{\Lambda (\omega )}{2}}{1+2\alpha {\left(\omega -\omega_{k}\right)}^{2}}$$

(10) $$\omega_{k}^{n+1}=\frac{\int_{0}^{\infty }\omega {\left|U_{k}\left(\omega \right)\right|}^{2}d\omega }{\int_{0}^{\infty }{\left|U_{k}\left(\omega \right)\right|}^{2}d\omega }$$

In Equations (9) and (10), $F\left(\omega \right)$, $U_{k}\left(\omega \right)$, and Λ(ω) represent the corresponding frequency domain expressions of f(t), $u_{k}(t)$, and λ(t), respectively.

### 2.3. Improved Transformer Time-Series Model for Gas Prediction

In the progression of driving faces, gas emission experiences marked volatility. Conventional time-series models, including ARIMA and SARIMA, which rely on autoregressive integrated moving averages, are often faltered in terms of interpretability and struggle to adequately identify anomalous data points within gas emission sequences. By integrating transformer models, a paradigm shift occurs, enabling the proficient learning of historical gas emission patterns and thereby facilitating forecasts and analyses of impending emission levels. To tailor transformer models for the prediction of gas emission time series, the seq2seq methodology is employed, enhancing their capacity to understand sequential dependencies. In this work, the machine learning model has been refined under the TensorFlow framework, bolstering its effectiveness in parsing and predicting complex temporal patterns inherent in gas emission datasets.

**Figure 2:**
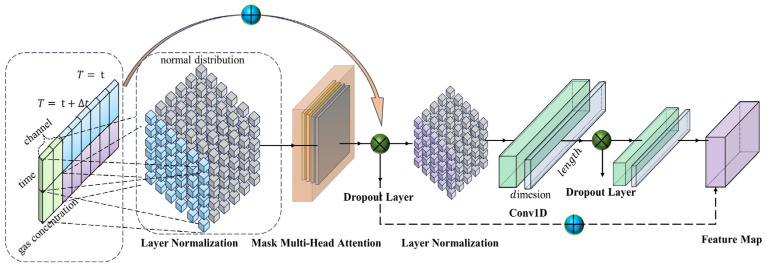
Transformer encoder structure applied to gas prediction time-series data.

Figure 2 showcases the structure of a transformer encoder adapted for predicting gas time-series data. Initially, the feature data undergo channel processing, where it is bifurcated into temporal data and gas concentration data. Subsequently, normalization is carried out on the feature data via a normalization layer to ensure uniformity in data preprocessing. The normalized data then engage in mask multihead attention, a mechanism designed to seize long-term dependencies and perform feature extraction, followed by regularization employing a dropout layer. Thereafter, the original feature data and the outputs from this processing stage are combined. Following these operations, layer normalization is administered to conduct additional normalization, enhancing the stability of the processing sequence. A ‘conv1d’ convolutional neural network is then introduced to further extract features pertinent to long dependency relationships, with another bout of regularization via a dropout layer subsequent to this step. Progressing further, a successive ‘conv1d’ convolutional neural network is employed to delve deeper into the extraction of long-range dependency features. Ultimately, the outcomes from the initial dropout layer and that of the second ‘conv1d’ convolutional neural network are aggregated, yielding the feature map—a synthesis of vital information for the predictive task at hand.

Figure 1 illustrates the sequential processing stages within the comprehensive transformer framework. The journey commences with the utilization of a cluster of n transformer encoders, tasked with meticulously extracting intricate feature maps from the input data. Subsequently, these feature maps undergo global average pooling, a strategic maneuver aimed at distilling salient feature representations while concurrently curtailing the propensity for overfitting, ensuring the model’s generalizability across varying data instances. The distilled features are then channeled into a suite of n multilayer perceptrons (MLPs), each meticulously configured with a rectified linear unit (ReLU) activation function. This inclusion of ReLU serves dual purposes: it introduces non-linearity into the model, a pivotal aspect for modeling complex relationships, and mitigates the vanishing gradient issue, wherein gradients become excessively small during backpropagation, threatening the learning efficacy of deep networks. Consequently, this strategic deployment of ReLU-augmented MLPs fortifies the neural network’s ability to learn intricate patterns, thereby enhancing its overall performance and predictive prowess.

The mathematical formulation underlying this model is delineated as follows: The model’s interface with the input data, represented as a tensor X of dimensions $\left(n_{timesteps},n_{features}\right)$, where $n_{timesteps}$ denotes the number of time steps and $n_{features}$ signifies the number of features per time step, initiates the process.

Initially, layer normalization is applied to the input data, as illustrated in Equation (11), yielding $X_{norm}$. This normalization step bolsters the model’s stability and enhances training efficiency. Next, Equation (12) embodies the multihead self-attention mechanism, a pivotal operation that enables the model to concurrently focus on disparate time step information. This capability is crucial for capturing intricate relationships inherent in time-series data, with the output being denoted as $X_{attn}$. Subsequently, residual connections accompanied by feedforward networks, as detailed in Equation (13), are employed. This integration not only facilitates the retention of important input data characteristics within the model but also tackles the vanishing gradient issue prevalent in deep networks. The output post this stage is labeled $X_{ffn}$. Finally, Equation (14) outlines the feedforward network processing, a stage dedicated to introducing further non-linear transformations to the output of the attention layers. This step amplifies the model’s expressive power, with the resultant processed data referred to as $X_{enc}$, thereby enriching the model’s capacity to model complex dependencies in the time-series data.

(11) $$X_{norm}=LayerNorm(X)$$

(12) $$X_{attn}=MultiHeadAttention(X_{norm},X_{norm},X_{norm})$$

(13) $$X_{ffn}=FFN(Layernorm\left(X+Xattn\right))$$

(14) $$X_{enc}=X+X_{ffn}$$

Upon completion of the aforementioned operations, Equation (15) introduces global average pooling, a dimensionality reduction step that consolidates information across the time dimension into a single, fixed-value representation. This operation, by averaging feature responses over time, extracts a summary statistic of the temporal information, yielding $X_{pool}$ as the condensed output, ready for subsequent analysis or decision-making tasks.

(15) $$X_{pool}=GlobalAveragePooling1D(X_{enc})$$

Specifically, Equation (16) denotes $X_{mlp}^{\left(i-1\right)}$ as the output from the preceding fully connected layer within the MLP stack, indicating the progressive transformation of features. Sequentially, Equation (17) presents $X_{drop}^{\left(i\right)}$ as the outcome after the Dropout operation, illustrating how the model prunes connections to enhance robustness and generalization.

This systematic application of MLP layers fortified with ReLU activations and Dropout forms a cornerstone of the model’s capacity to navigate and model the high-dimensional complexity inherent in the relationship between inputs and the desired predictions.

(16) $$X_{mlp}^{\left(i\right)}=ReLU(Dense\left(X_{mlp}^{\left(i-1\right)}\right))$$

(17) $$X_{drop}^{\left(i\right)}=Dropout(X_{mlp}^{\left(i\right)})$$

Culminating the series of stacked MLP layers is the final fully connected layer, encapsulated in Equation (18), which aggregates the learned features to produce the model’s predictions. Here, n denotes the total count of MLP layers within the architecture, underscoring the depth of feature extraction and transformation preceding this ultimate stage. The output, symbolized as $\hat{Y}$, represents the model’s predicted values, embodying the distilled knowledge from the intricate interplay of input features through multiple layers of non-linear transformations. This concluding layer bridges the gap between the abstract feature representations honed by the preceding MLPs and the tangible, real-world predictions the model is tasked with delivering.

(18) $$\hat{Y}=Dense(X_{drop}^{\left(n\right)})$$

### 2.4. Error Analysis of Extreme Gradient Boosting Regressor Predictions

The Extreme Gradient Boosting Regressor, or XGBoost Regressor (XgbRegressor), represents an advanced learning algorithm rooted in the gradient boosting paradigm. Its fundamental principle revolves around assembling a robust ensemble from simpler, less accurate models, commonly referred to as weak learners [26]. Within the scope of this research, the XgbRegressor is instrumental in conducting an in-depth error analysis, where it scrutinizes the outcomes generated by the transformer model [27]. This analytical endeavor aims to refine prediction accuracy and enhance understanding of the model’s performance.

Depicted in Figure 1 is the systematic procedure for evaluating prediction inaccuracies. The XgbRegressor is strategically deployed to quantify the discrepancy between the model’s forecasted values and the true observations, serving a dual purpose. Not only does it critically appraise the current prediction errors, but it also harnesses this analysis to make informed projections about error tendencies in the upcoming time steps.

### 2.5. Dimensional Analysis for Establishing Real-Time Relative Gas Emission Volume

Data preceding the advancing driving face remain uncertain, and in practical excavation scenarios, data gathering must not hinder the progress of the driving faces. An overabundance of parameter types being measured can inadvertently slow the advancement rate, disrupting normal operational efficiency.

Parameters pertinent to gas emissions at the driving face are primarily determined by on-site conditions, with a primary requirement being ease of acquisition and measurement. Yaolin Cao [28] emphasizes that ventilation volume plays a pivotal role in influencing methane distribution and dilution within mines, thereby affecting overall gas concentrations and potentially fostering accumulations that could lead to outbursts. Methane concentration, indicative of methane content in air, is a paramount factor in assessing the explosivity of mine atmospheres. The consideration of advancement length is imperative because, as the coal seam surface is newly exposed during excavation over increasing distances, it impacts the release of adsorbed methane and fosters emergent zones of potential instability. The unit time gas emission volume reflects the rate at which methane is discharged into the mine atmosphere, serving as a critical indicator for anticipating shifts in methane release patterns that might precipitate sudden events.

Thus, for the driving face, the focus is on gathering readily accessible data. Under the principle of differentiated processes, dimensional analysis is conducted on key parameters such as airflow, gas concentration, progression distance, and instantaneous gas emission volume. This analytical approach aids in establishing a real-time, relative measure of gas emission volume.

(19) $$m_{l}=\frac{m^{3}}{t}=\frac{lA\rho }{\Delta t}$$

(20) $$q_{x}=\frac{m^{3}\times n}{\Delta t}$$

(21) $$q_{r}=\frac{q_{x}}{m_{l}}=\frac{m^{3}\times n}{\Delta t}\times \frac{\Delta t}{lA\rho }=\frac{m^{3}}{kg}$$

First, calculate the unit time coal falling quantity $m_{l}$ using Equation (19), and calculate the relative gas emission quantity $q_{x}$ using Equation (20). Through dimensional analysis, using Equation (21) obtain the final result of the real-time relative gas emission volume $q_{r}$. In the equations, $q_{r}$ represents the real-time relative gas emission volume, $m^{3}/kg$; $q_{x}$ represents the relative gas emission volume, $m^{3}/t$. $m_{l}$ represents the unit time coal falling quantity, t. l represents the driving length, m. Δt represents the time interval of coal cutting, min; n represents the gas concentration in the driving face in percentage, %; ρ represents the density of coal, $kg/m^{3}$.

### 2.6. Persist Anomaly Detection for Gas Abnormal Emission Monitoring

In the realm of coal and gas outburst incidents at driving faces, where data availability is often sparse or unreliable, implementing semi-supervised or supervised monitoring methodologies for anomaly detection presents significant challenges. To tackle this issue, the Persist Anomaly Detection (PersistAD) algorithm emerges as an unsupervised solution tailored for time-series data, adept at autonomously filtering out recurring patterns such as seasonality and trends in gas emission sequences. By meticulously tracking abrupt deviations in gas emission patterns over sustained intervals, PersistAD assumes a pivotal role in identifying anomalies within these critical datasets.

At its core, PersistAD relies on a dual rolling aggregation strategy, a mechanism that consolidates data from two time spans for thorough analysis. This methodology is indispensable for deciphering and projecting trends and cyclical behaviors intrinsic to time-series data. Within the PersistAD framework, this dual aggregation process identifies instances of data deviation across two distinct time segments, flagging these segments as potential anomalies.

**Figure 3:**
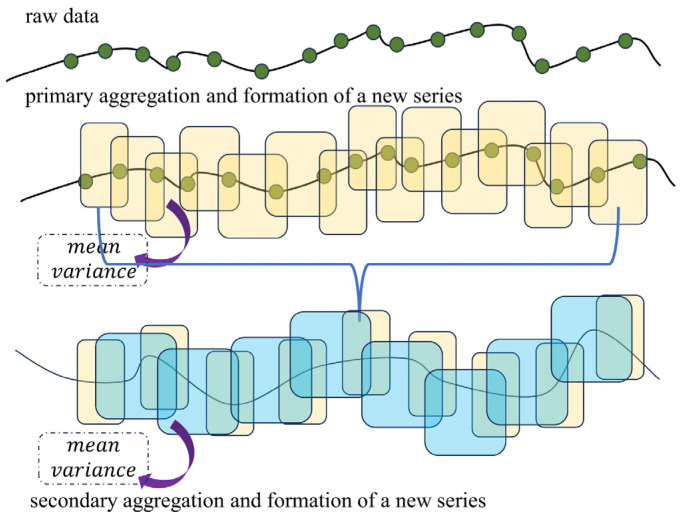
Double rolling aggregation method illustration.

Figure 3 visually elucidates the mechanics of this dual rolling methodology. Here, the “raw data” represents the unprocessed dataset, while the yellow translucent rectangles depict the primary aggregation tier, encapsulating key statistical summaries such as mean and variance. These statistics then form the foundation of the first sequence. Subsequently, a secondary aggregation is performed, giving rise to the second sequence, visually depicted by green rectangles.

### 2.7. Details the Arrangement and Deployment Strategy for Sensors at the Driving Face

The layout of sensors at the driving face has a direct impact on the accuracy of methane concentration monitoring, with a primary aim to detect methane accumulation and prevent explosions. In the selected mine for this study, sensor placement follows a strategic design. The T1 sensor is positioned 5 m away from the driving face, passively monitoring methane emissions and advancing alongside the progress of the work front [29]. Its role is crucial in tracking the volume of methane emitted.

Meanwhile, the T2 sensor is strategically installed 28 to 30 m distant from the working face, focusing on detecting whether wind velocity and methane concentrations in the return air flow exceed predefined thresholds. Similarly, the T3 sensor, stationed at the entrance of the excavation tunnel, actively monitors for any exceedances in wind velocity and methane concentration within the tunnel itself, thereby providing a comprehensive surveillance of these critical parameters.

**Figure 4:**
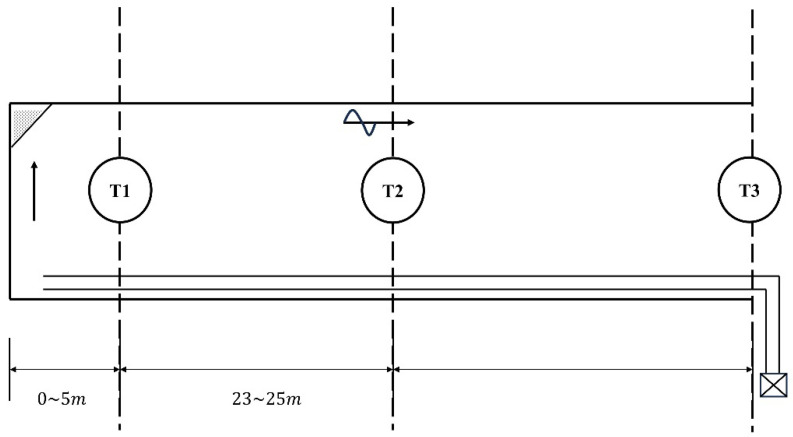
Schematic diagram of sensor layout at the driving face.

Figure 4 illustrates a schematic diagram of this sensor deployment at the driving face. Depicted at the bottom of the figure is the local fan, integral to ventilation. The layout visualizes the T1 sensor precisely located 5 m from the work interface, the T2 sensor situated strategically between 28 and 30 m from the face, and the T3 sensor, marking its position at the entry of the excavation drift.

## 3. Results and Discussion

Upon conclusion of the methodology overview, this study proceeds with computational simulations executed in a Jupyter environment, leveraging Python version 3.10.13. The current experiment has selected a mine from Shaanxi, China for testing, with a total of three driving faces data. The implementation hinges upon the robust foundations of the TensorFlow framework for machine learning capabilities, numpy for efficient scientific computations, and Optuna for sophisticated hyperparameter optimization. The confluence of these technologies within our experimental setup underscores a rigorous and adaptive approach to model development and evaluation. The trial outcomes affirmatively illustrate the practical applicability and effectiveness of the proposed methods. The successful integration within this computational framework attests to the method’s robustness and its capability to deliver reliable insights under diverse scenarios. In Appendix A, we provide additional analysis of the codes to support our findings.

### 3.1. Reliability Test of the AN Normalization Method

**Figure 5:**
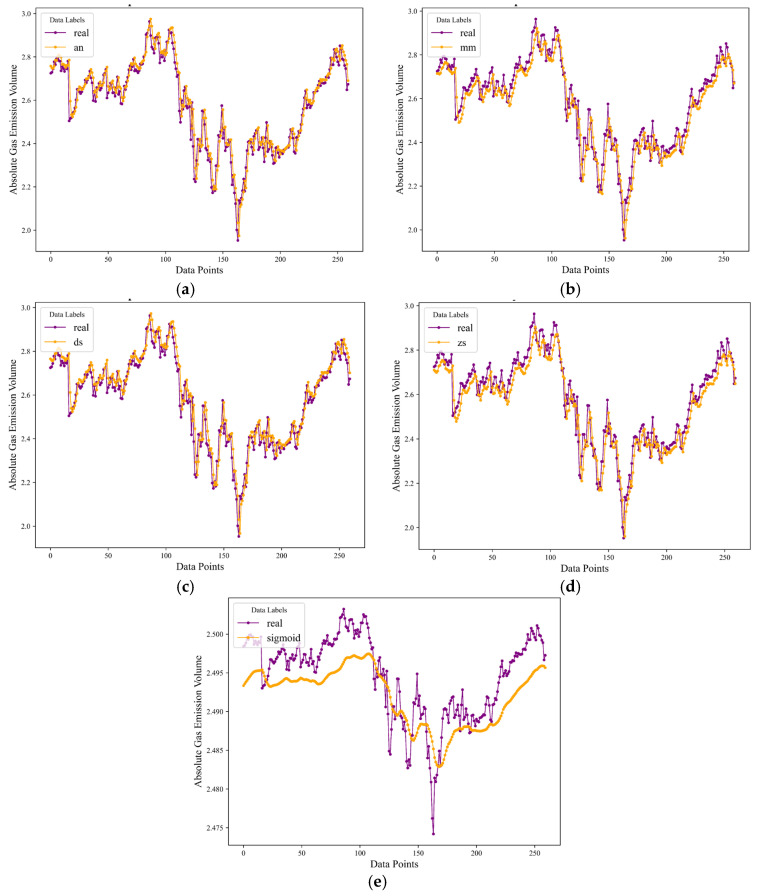
Comparison chart of prediction values between AN normalization and other normalization methods. (**a**) adaptive normalization method validation results. (**b**) min-max normalization method validation results. (**c**) decimal scaling method validation results. (**d**) z-score method validation results. (**e**) sigmoid method validation results.

Throughout this experimental investigation, the adaptive normalization (AN) strategy was implemented to preprocess the gas emission dataset. The dataset was partitioned into non-overlapping sliding windows of size 5, with a scaling factor (k-value) set at 1, as a precursor to the standardization process. Subsequently, a comparative analysis was conducted between AN and several alternative normalization techniques, specifically min–max (MM), Decimal Scaling (DS), Z-Score (ZS), and Sigmoid (SD), all within the framework of a Gated Recurrent Unit (GRU) model. The predictive assessment encompassed 260 data subsets. The visual representation of these findings is encapsulated in Figure 5, where: Figure 5a depicts the forecasts generated using AN normalization, Figure 5b showcases the outcomes derived from MM normalization, and Figure 5c–e correspond to the predictive results achieved with DS, ZS, and SD normalization techniques, respectively. From this comparative analysis, AN normalization emerged as the superior approach, demonstrating the finest predictive performance. In contrast, the SD normalization method yielded the least favorable results. A meticulous quantitative evaluation, presented in Table 1, further validates AN’s leading position. Across metrics such as Mean Squared Error (MSE), Root Mean Squared Error (RMSE), Mean Absolute Error (MAE), Relative Mean Error (RME), and the coefficient of determination (R^2^), AN consistently outperformed its counterparts. Most notably, the AN method attained an impressive R^2^ value of 0.914624, underscoring its exceptional ability to explain the variability in the data and affirming its superiority in enhancing the GRU model’s predictive accuracy.

**Figure 6:**
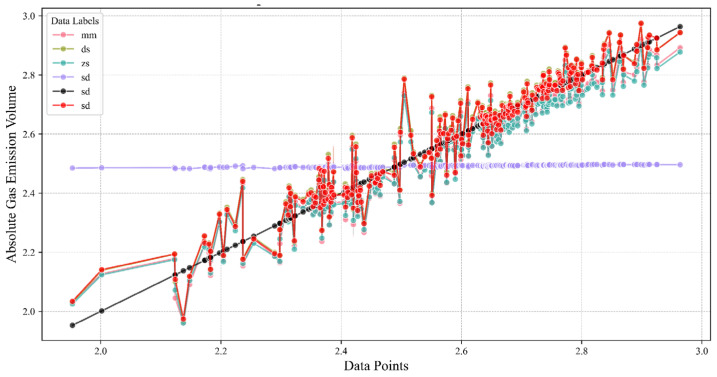
Comparison chart of prediction errors.

The comprehensive comparison among the normalization techniques is visually depicted in Figure 6. Owing to its notably lower error magnitude, the representation of the AN method is somewhat overshadowed by the traces of other methodologies, barely distinguishable yet subtly protruding above them. In this graphical depiction, the AN method is distinguished by a brown dashed line, symbolizing its minimized deviation from the actual data trajectory. Conversely, the original dataset is traced by a prominent black solid line, serving as the benchmark against which normalized data performances are gauged. The SD method, conversely, stands out with the most substantial discrepancy, manifesting the highest error amongst the considered approaches. The remaining normalization techniques exhibit error magnitudes that bridge the gap lying between the tightly controlled errors associated with the AN method and the more conspicuous errors characterizing the SD method, thereby painting a holistic picture of comparative performance within this study.

### 3.2. Preprocessing of Gas Emission Time-Series Data

This research encompasses a comprehensive dataset comprising 1460 instances of gas emission data. Among these, the initial 1200 records serve as the training dataset, while the subsequent 260 entries are reserved for predictive modeling. Prior to further analysis, the dataset undergoes standardization using the adaptive normalization (AN) technique, which confines its values within the interval of −1 to 1. As part of the preprocessing stage, statistical quartile analysis is utilized to identify and exclude outliers, specifically by employing the first and third quartiles.

**Figure 7:**
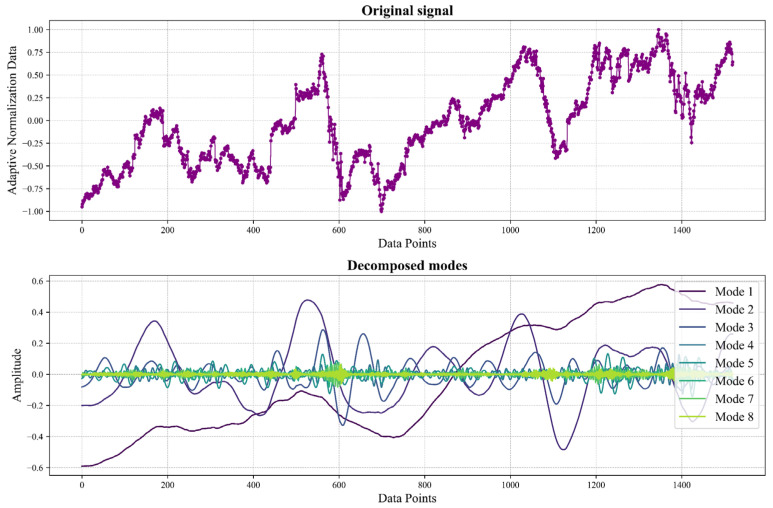
VMD decomposition plot.

Following standardization, an Enhanced Empirical Mode Decomposition (EEMD)-guided Variational Mode Decomposition (VMD) is employed for a more in-depth data analysis. Through EEMD, a decomposition level of 9 is determined to be optimal, while VMD is parameterized with settings including α=2000, τ=0, DC component removal (DC=0), initialization mode (init=2), and a tolerance level of $1\times 10^{-7}$. This adaptive decomposition, with its 9-component structure, is visually depicted in Figure 7. Here, the “original signal” represents the dataset post AN standardization, while the various “decomposed models” illustrate the individual components obtained from the VMD process.

**Figure 8:**
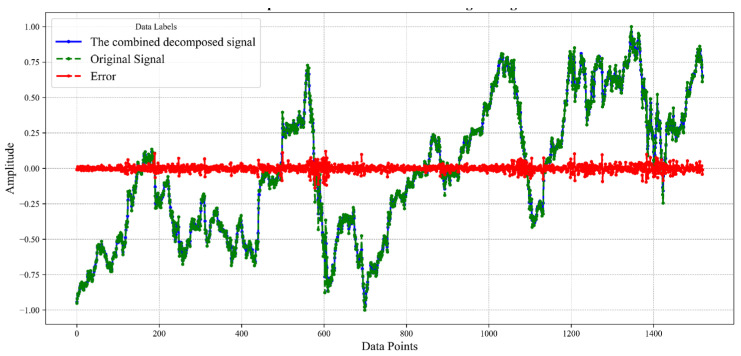
Comparison of reconstructed and original signals.

To evaluate the accuracy of this decomposition, Figure 8 presents the decomposition error. In this representation, “the combined decomposed signal” is derived from the summation of all VMD components, closely mirroring the “original signal” from AN standardization, indicating a high degree of overlap. The term “Error” quantifies the disparity between the reconstructed signal and the original data, with values oscillating roughly between 0.14 and −0.18. Upon meticulous calculation, the extremities of error are quantified, with the maximum error reaching 0.135 and the minimum dipping to −0.177. The narrow range of these error margins attests to the efficacy and precision of the EEMD-VMD decomposition strategy, confirming its suitability for the task at hand.

### 3.3. Optimizing Transformer Model Hyperparameters with Optuna

Optuna is an open-source Python library designed to automate the intricate process of hyperparameter optimization. It finds broad application across a spectrum of machine learning and deep learning domains, spanning from image classification and natural language processing to recommendation systems, among numerous others. By employing advanced statistical techniques, Optuna intelligently assesses the impact of individual parameters on model performance, thereby enabling efficient tuning of multiple parameters simultaneously. This automated approach not only accelerates the model development cycle but also enhances model accuracy and robustness by discovering optimal configurations in complex model architectures. Its versatility and efficiency make Optuna a powerful tool in the arsenal of data scientists and machine learning engineers seeking to maximize the potential of their models.

Within the scope of this experimentation, the hyperparameters of the transformer model underwent refinement through a rigorous process of 100 iterations, facilitated by Optuna. Integrating seamlessly with the TensorFlow ecosystem, Optuna was configured to incorporate pruning callbacks—a mechanism to halt unpromising trials early—and model checkpoint callbacks, which preserved the model state at each epoch, ensuring the preservation of the best performing model iteration thus far. The metric of choice for guiding this optimization quest was the Mean Squared Error (MSE), a pivotal indicator of prediction accuracy. Harnessing Optuna’s capabilities, a refined transformer model emerged after 50 iterations, with its learning regimen fueled by the predictions derived from the VMD-decomposed data components. A meticulous selection of eight key hyperparameters for the transformer model was subject to this optimization endeavor, as detailed in Table 2.

**Figure 9:**
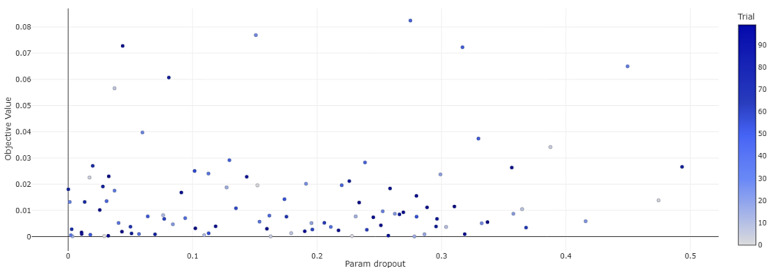
Optuna hyperparameter optimization dropout parameter 100 training results graph.

**Figure 10:**
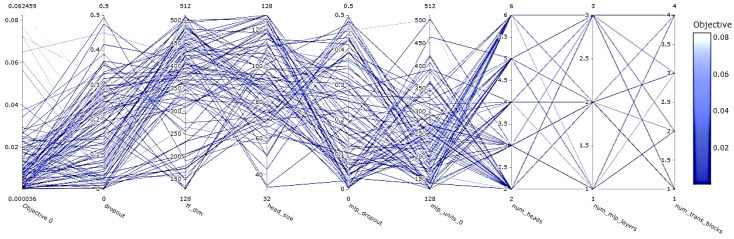
Optuna hyperparameter optimization 100 training iterations graph.

Depicted in Figure 9 is a visual representation highlighting the dropout rates that correlated with the minimal MSE values observed throughout the training epochs, exemplifying how Optuna explores the solution space for each parameter. Analogous graphical illustrations are provided for the remainder of the tunable parameters. Further consolidating this narrative, Figure 10 encapsulates the overarching trajectory of the hyperparameter optimization training, specifically focusing on the tuning journey involving dropout, feed-forward dimension (ff_dim), head size, MLP dropout, MLP units, number of attention heads (num_heads), number of MLP layers (num_mlp_layers), and the quantity of transformer blocks (num_trans_blocks). The optimization process achieved a remarkable minimum objective value of 0.000036, testifying to the model’s significantly enhanced predictive capability following optimization. A full summary of the calibrated parameter ranges, crucial for attaining this level of performance, is conveniently compiled in Table 2.

Figure 10 offers a visual summary of the hyperparameter optimization outcomes, mapping the progression of selected parameter values against their associated Mean Squared Error (MSE) scores across the full span of 100 training iterations. This graphical portrayal facilitates a clear understanding of how each parameter’s tuning contributes to the minimization of the MSE, thereby elucidating the optimization journey.

**Figure 11:**
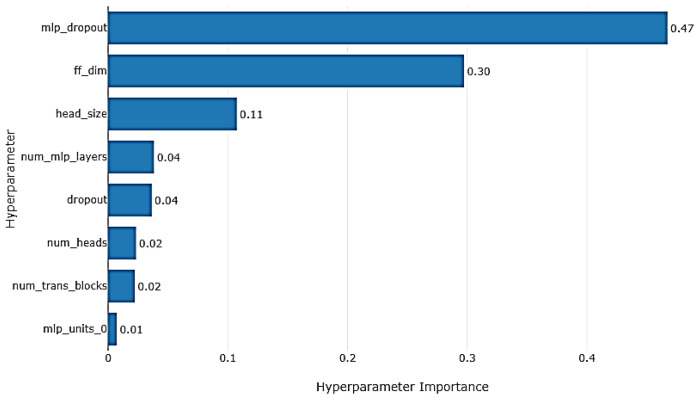
Optuna hyperparameter optimization importance plot for 100 training iterations.

Further enriching the analysis, Figure 11 delves into the relative significance of the hyperparameters, presenting a comparative assessment of their individual impacts on model performance. By quantifying the importance of each hyperparameter, this feature of Optuna empowers researchers to discern which factors are most influential in refining the model’s predictive accuracy.

### 3.4. Time-Series Hybrid Prediction Model for Gas Emission Forecasting

**Figure 12:**
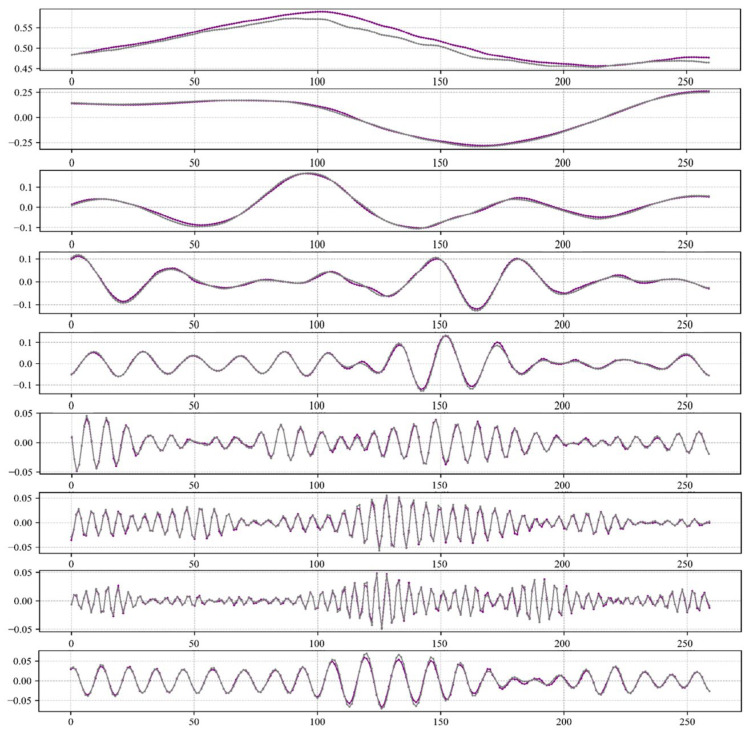
Transformer predicted decomposed variable results graph.

Upon completion of the Variational Mode Decomposition (VMD) for individual signal components, a meticulous optimization of the transformer model’s parameters was undertaken to derive conclusive outcomes. Leveraging the refined parameter configurations, forecasts for the subsequent 260 time steps were generated. The efficacy of these predictions is encapsulated in Figure 12, where a striking correspondence between the projected values for each decomposed signal and the authentic dataset is evident, attesting to the model’s performance. Progressing sequentially down the figure, each subplot juxtaposes the forecasted trajectories (in purple) against the actual data points (in gray), highlighting a high degree of synchronization. The purple line represents the predicted values, and the gray line represents the actual values.

**Figure 13:**
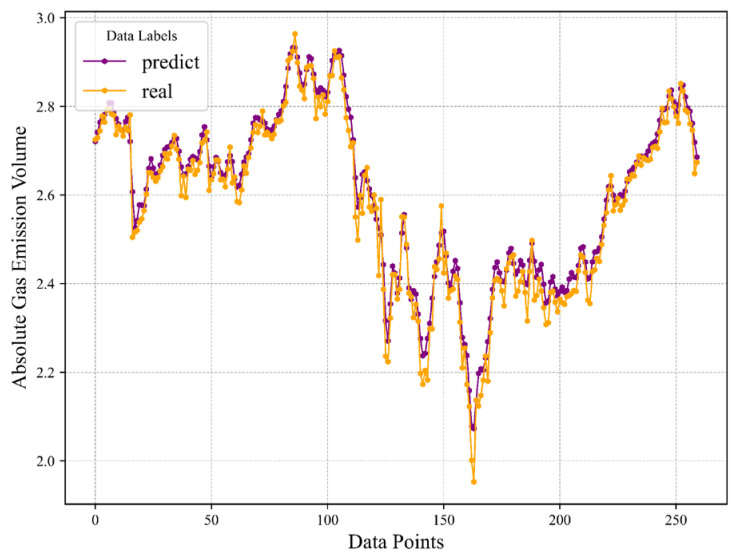
Comparison graph of predicted results and real data for the hybrid prediction model.

Supplementing this analysis, Figure 13 introduces an additional layer of scrutiny by showcasing the post hoc estimation of discrepancies between the genuine and predicted values, augmented with the integration of the XGBoost regressor (xgbregressor). The overlapping parts indicate that the effects are the same at this point. This step serves to refine error quantification, offering a nuanced perspective on the prediction accuracy. Table 3 consolidates the terminal error metrics, evidencing exceptional predictive prowess. Remarkably, the coefficient of determination (R^2^) approximates 0.98.

**Figure 14:**
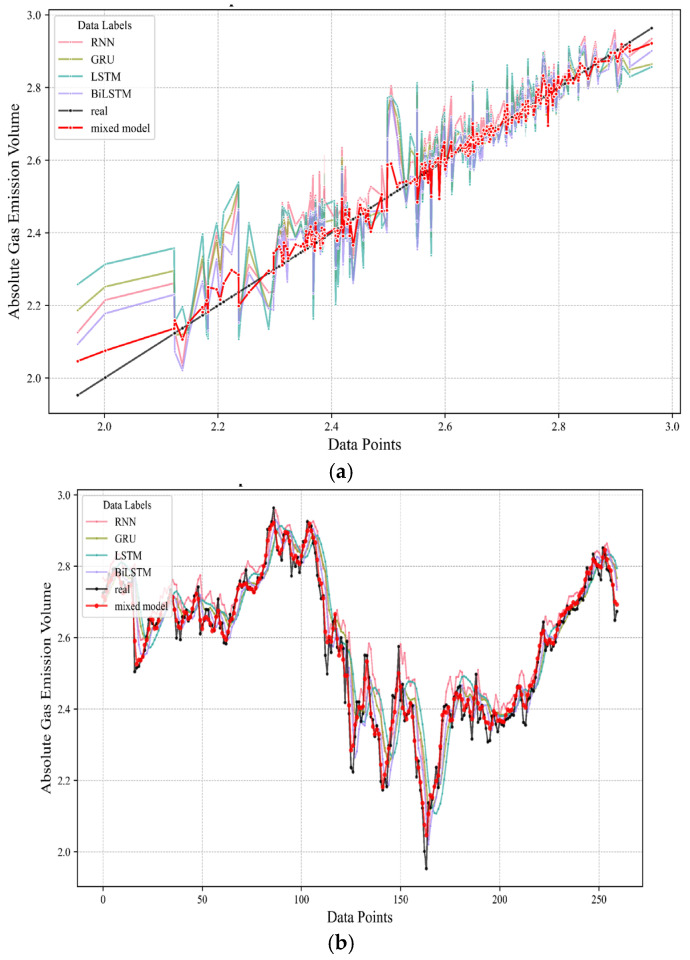
Comparison of predicted results from multiple algorithms with real data. (**a**) diagonal error plot between different algorithms. (**b**) temporal evolution plot of prediction results between different algorithms.

Upon the conclusion of the forecasting stage, a comparative assessment was conducted between the proposed model’s predictions and those generated by conventional algorithms. This comparative analysis is visually encapsulated in Figure 14. Figure 14a presents a diagonal error plot, where the proximity of each colored dashed line to the black reference line symbolizes a minimized prediction error. Each line represents the outcome of a different predictive algorithm. Of particular note, the “mixed model,” which signifies the hybrid forecasting model integrating temporal concentration dynamics, emerges with the tightest alignment to the black line, indicative of its superior performance in terms of minimizing prediction deviations from actual values. Expanding upon this visual narrative, Figure 14b offers another perspective, depicting the temporal evolution of prediction accuracy for individual data points. This chronological display accentuates the performance consistency and accuracy of the models over successive time intervals, further validating the “mixed model’s” outstanding ability to consistently approximate actual emission patterns across varying timeframes. Collectively, these visualizations in Figure 14 affirm the heightened predictive power and temporal adaptability of the hybrid model in comparison to traditional methodologies.

### 3.5. Verification of the Results from the Remaining Driving Faces

**Figure 15:**
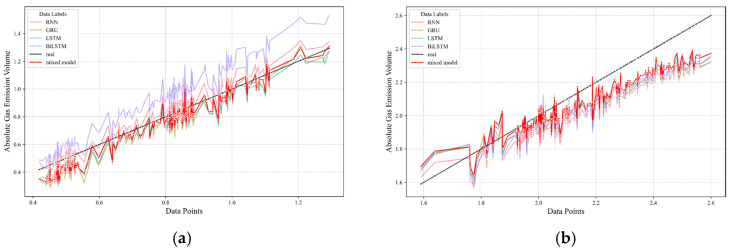
Diagram illustrating prediction error for additional driving faces. (**a**) the diagonal error plot of the first driving face. (**b**) the diagonal error plot of the second driving face.

**Figure 16:**
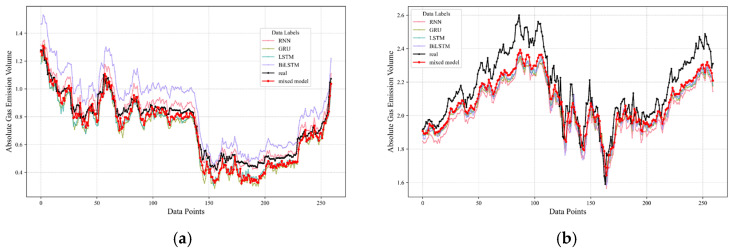
Chart displaying actual values versus predicted for other driving faces. (**a**) the prediction results plot of the first driving face. (**b**) the prediction results plot of the second working face.

On this foundation, predictions were extended to encompass distinct datasets from two additional driving faces, with an aggregate forecast performance proving satisfactory. These outcomes are visually depicted in Figure 15 and Figure 16, wherein the actual data are traced by a solid black line, while the model-generated predictions are illustrated using a standout red line, delineating the forecasted trend.

In these illustrations, Figure 15a and Figure 16a are devoted to the first of the alternative driving faces. Following this, Figure 15b and Figure 16b redirects attention to the second driving face, further substantiating the wide-ranging applicability and reliability of the predictive framework across a variety of operational contexts within the mining environment. Through these visual presentations, the effectiveness of the model in predicting gas emissions for datasets that have not been modeled previously is convincingly demonstrated, highlighting the robustness and generalizability of the adopted methodology.

### 3.6. Unsupervised Time-Series Warning Model

Following the prediction of gas concentration sequences, a Bootstrap algorithm is utilized to resample the forecasted outputs with repetition, leading to the derivation of the ultimate prediction confidence interval, as documented in the literature [30,31,32]. Relying on the mean value extracted from the terminal instant, a real-time approximation of the relative gas emission volume is computed. This computation forms the groundwork for the employment of the PersistAD anomaly detection algorithm, which is strategically tasked with surveilling abrupt amplifications, indicative of potential anomalies potentially linked to distinctive geological features or irregular breaches in coal faces.

**Figure 17:**
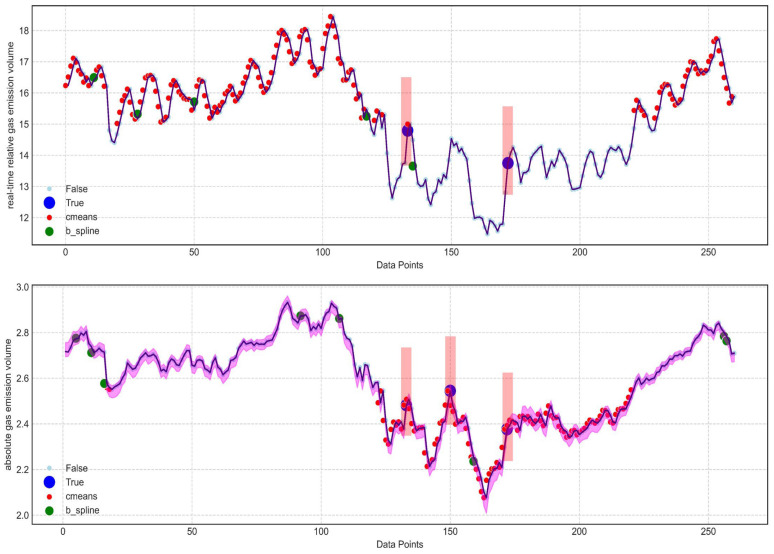
Real-time relative gas emission volume and absolute gas emission volume mutation point detection graph.

The visual representation of these findings is exhibited in Figure 17, wherein red rectangular bars denote anomaly points, aligning with corresponding blue data points, symbolizing anomalies. Conversely, cyan points symbolize periods of regular emission activity. The sequence commences with the first image illustrating anomalies identified in the real-time monitoring of relative gas emission volumes, succeeded by a display of anomalies detected within the ambit of absolute gas emission volume surveillance. Superimposed over these visuals, the pink areas denotes the confidence interval meticulously approximated through the Bootstrap algorithm, furnishing a pivotal gauge of the prediction variability and reliability. This holistic strategy, entwining advanced forecasting methodologies with vigilant anomaly surveillance, bolsters the system’s resilience against unforeseen occurrences in subterranean mining activities. Simultaneously contrasted with various anomaly detection algorithms, in the depicted figure, red dots represent anomalies detected via fuzzy C-means clustering [33], while green dots denote anomalies monitored through b-splines regression [34]. The range of detection by the fuzzy C-means clustering algorithm is noted to be excessively dense. Although the performance of the green dot methodology is somewhat superior, it fails to accurately capture peak values and sudden fluctuations in gas emissions, which are critical indicators for anomaly monitoring. The three-dimensional renkey approximation algorithm [35], due to its requirement for excessively high dimensional data, has not been included in this discussion.

## 4. Conclusions

This paper proposes a hybrid model for time-series prediction and an unsupervised method for time-series anomaly detection. Innovatively, in terms of time-series prediction, it combines adaptive normalization with transformer encoder, conv1d, and mlp, effectively improving the prediction accuracy and performance of neural networks. In terms of anomaly detection, a new metric, real-time relative gas emission volume, is proposed for gas emission, which effectively reduces the lag of warning indicators for mining faces. When there is no effective data for gas outburst, an unsupervised monitoring method is proposed, providing a new monitoring method for practical work in coal mine driving faces.

Computer experimental methods and theoretical analyses are employed in this research to study gas prediction and gas anomaly warning in mining faces. The main research findings are as follows:

(1) A hybrid time-series prediction model is proposed, which innovatively preprocesses data using adaptive normalization and adopts the EMA method, making the data closer to the gas emission pattern of mining faces. The EEMD + VMD method is employed to decompose data, addressing the issue of determining the order of VMD.
(2) The transformer architecture is improved by incorporating encoder, multiple conv1d layers, and mlp units. Parameter optimization is conducted using the optuna framework, and xgbregressor is used to estimate errors. Experimental results show that the prediction model can accurately forecast future gas emissions.
(3) The confidence interval of the prediction results is estimated using bootstrap sampling, and a dimension analysis is conducted to obtain a more practical indicator, real-time relative gas emission volume. The PersistAD anomaly detection method is used to detect abrupt points, addressing the issue of insufficient data in gas outburst monitoring.

This study introduces a gas outburst monitoring method for coal mines, aiming to swiftly respond to such events and minimize safety risks. Future research focuses on enhancing real-time monitoring via theoretical and microseismic analyses, integrating transformers with cutting-edge deep learning for improved, timely gas emission predictions. Efforts will also optimize data collection and feature extraction for better model performance and explore model interpretability. The goal is to incorporate this advanced predictive system into mine safety infrastructure, enabling rapid, informed responses and enhancing coal mining safety standards.

## Figures and Tables

**Table 1:** Comparison table of errors between AN normalization and other normalization methods.

	AN-GRU	MM-GRU	DS-GRU	ZS-GRU	SD-GRU
MSE	0.003444	0.005877	0.003693	0.004094	0.000015
RMSE	0.0586585	0.063103	0.060768	0.063986	0.003836
MAE	0.041230	0.049664	0.043391	0.051743	0.003390
RME	0.015660	0.18863	0.016481	0.019653	0.001358
*R* ^2^	0.914624	0.901287	0.908458	0.898505	0.489572

**Table 2:** Table of range of values selected for transformer hyperparameter optimization.

head_size	num_heads	ff_dim	num_trans_blocks
32~128	2~6	128~512	1~4
num_mlp_layers	mlp_units	dropout	mlp_dropout
1~3	128~512	0~0.5	0~0.5

**Table 3:** Error between predicted results and real values for the hybrid model.

MSE	RMSE	MAE	RME	*R* ^2^
0.000149	0.000767	0.027702	0.007623	0.980975

## Data Availability

The data are not publicly available due to commercial confidentiality, as they contain information that could compromise the privacy of research participants.

## Appendix A

The code will be upload in github https://github.com/3192575523/gas_ourburst_prediction (accessed on 13 May 2024).
